# Electrically facilitated translocation of protein through solid nanopore

**DOI:** 10.1186/1556-276X-9-140

**Published:** 2014-03-24

**Authors:** Lingzhi Wu, Hang Liu, Wenyuan Zhao, Lei Wang, Chuanrong Hou, Quanjun Liu, Zuhong Lu

**Affiliations:** 1 State Key Laboratory of Bioelectronics, Southeast University, Nanjing 210096, China; 2 School of Geography and Biological Information, Nanjing University of Posts and Telecommunications, Nanjing 210046, China

**Keywords:** Protein translocation, Solid state nanopore, Current blockage, Translocation time

## Abstract

Nanopores have been proven as versatile single-molecule sensors for individual unlabeled biopolymer detection and characterization. In the present work, a relative large nanopore with a diameter of about 60 nm has been used to detect protein translocation driven by a series of applied voltages. Compared with previous studied small nanopores, a distinct profile of protein translocation through a larger nanopore has been characterized. First, a higher threshold voltage is required to drive proteins into the large nanopore. With the increase of voltages, the capture frequency of protein into the nanopore has been markedly enhanced. And the distribution of current blockage events is characterized as a function of biased voltages. Due to the large dimension of the nanopore, the adsorption and desorption phenomenon of proteins observed with a prolonged dwell time has been weakened in our work. Nevertheless, the protein can still be stretched into an unfolded state by increased electric forces at high voltages. In consideration of the high throughput of the large nanopore, a couple of proteins passing through the nanopore simultaneously occur at high voltage. As a new feature, the feasibility and specificity of a nanopore with distinct geometry have been demonstrated for sensing protein translocation, which broadly expand the application of nanopore devices.

## Background

Over past decades, nanopores have been widely evolved in various devices for investigating unlabeled biopolymers at the single-molecule level [1,2]. Although the focus is on nucleic acids, proteins are becoming a prime target for investigation [3,4]. Protein transport through the cellular compartments is a very important physiological process for substance and energy metabolism of living cells [5-7]. Compared with DNA sequencing, protein translocation through nanopores is more challenging. First, proteins have a variety of charge profiles depending on the solvent environment. When pH is lower than the isoelectric point of proteins, the net charge of protein is positive, while the reverse case is negatively charged [8,9]. Second, each protein has a unique structural architecture, including the primary peptide chain, secondary, tertiary, and quaternary structures, which are responsible for their biological functions. Yet the native protein conformation is only marginally stable. Once the protein’s physical and chemical environment is modestly changed, the rigid structure of a protein will unfold into random coils [8,10]. These features of proteins are distinct from the linear DNA with a uniform negative charge. Thus, nanopore experiments on proteins are more complicated than the DNA sequencing.

Yet for all that, a set of experiments have demonstrated the unique and advantageous ability of nanopores to discriminate protein translocations [9-14], protein folding [10,13,15-18], and enzymatic kinetic reactions [19-26] in the context of single-molecule analysis. For example, nanopores have been used to discriminate the surface charge and size of proteins as a function of pH [27-29]. The unfolding transition and structural stability of proteins have also been studied by chemical and thermal denaturation, as well as electric field stretching [3,10,13,15,30]. An interesting phenomenon is non-specific adsorption interaction between proteins and nanopores, whereby the protein sticks to the pore for a prolonged dwell time [18,31,32]. These results closely depend on the quality and geometry of the nanopores used, most of which focus on the small nanopores with the dimension comparable to the analyzers to achieve an optimal solution. Even so, the capture rate of proteins is low in nanopore experiments, and the electroosmotic flow against electrophoretic mobilities of proteins through silicon nitride membranes is dominant in small nanopores [9,10,18,27,33,34]. Meanwhile, the adsorption interaction of proteins easily makes the small pore plugged [31,32]. Therefore, to reduce these negative effects, nanopores with a larger scale are an alternative choice to analyze the varied targets. First, the arriving probability of protein in pore mouth is governed by free diffusion in bulk, which is referred to the pore geometry [9,35]. A higher capture rate is expected for large nanopores [35]. And both electroosmotic effect and protein-pore interaction corresponding to the electric double layer along the charged inner wall will be weakened in large nanopores; thus, more proteins will freely pass through nanopores [36,37]. Additionally, more space in large nanopores is in favor of the surface modification to change the physical and chemical properties of pores [38,39], which will broadly expand the utility of nanopores for biological sensing. Certainly, the signal-to-noise ratio of the blockade current will inevitably deteriorate if the pore is too large. Hence, the choice of nanopore with a suitable dimension is critical for the design of nanopore devices to understand the physical mechanism of molecules translocating through nanopores.

Herein, bovine serum albumin (BSA), an important transport protein, is chosen to pass through a silicon nitride nanopore with a diameter of 60 nm. By applying a set of biased voltages, the protein swims through the large channel with a detectable signal-to-noise ratio of the blockage current. Comparing with small nanopores, a higher threshold voltage of 300 mV is observed to drive the protein into the nanopore. With the voltage increasing, the current blockage events are greatly enhanced and are classified as a function of voltages. At the medium-voltage region, the amplitude of blockage current increases linearly while the dwell time decreases exponentially with the increasing voltage. Despite more free space in our large nanopore, the adsorption and desorption phenomenon of proteins has also been detected with a prolonged dwell time, but it is greatly weakened compared with small nanopore cases. With further increasing voltage, the protein is more likely to be destabilized by the applied electric forces. And a couple of proteins can pass through the nanopore simultaneously. Together, the experiments yield a new aspect of protein transport through a solid-state nanopore with a large scale. The results may help in the future development of nanopore devices such as single-molecule sorting, dynamic molecular interaction, and controllable self-assembly of molecules.

## Methods

### Chemicals and materials

Pure (>98%) crystallized BSA from Fraction V was purchased from Sigma-Aldrich (St. Louis, MO, USA) and used without further purification. All other chemical reagents used in our experiment were of analytical grade without further purification. All samples were prepared by Milli-Q super purified water with resistance >18 MΩ/cm (Millipore, Billerica, MA, USA). All solutions were filtered with 0.02-μm Anotop filter (Whatman, Maidstone, UK) before using. Nanopores were hydrated with the addition of degassed and filtered KCl electrolyte solution buffer. Electrolyte strength was typically 1 M/1 M KCl *cis*/*trans* in protein translocation studies.

### Nanopore fabrication

**Figure 1:**
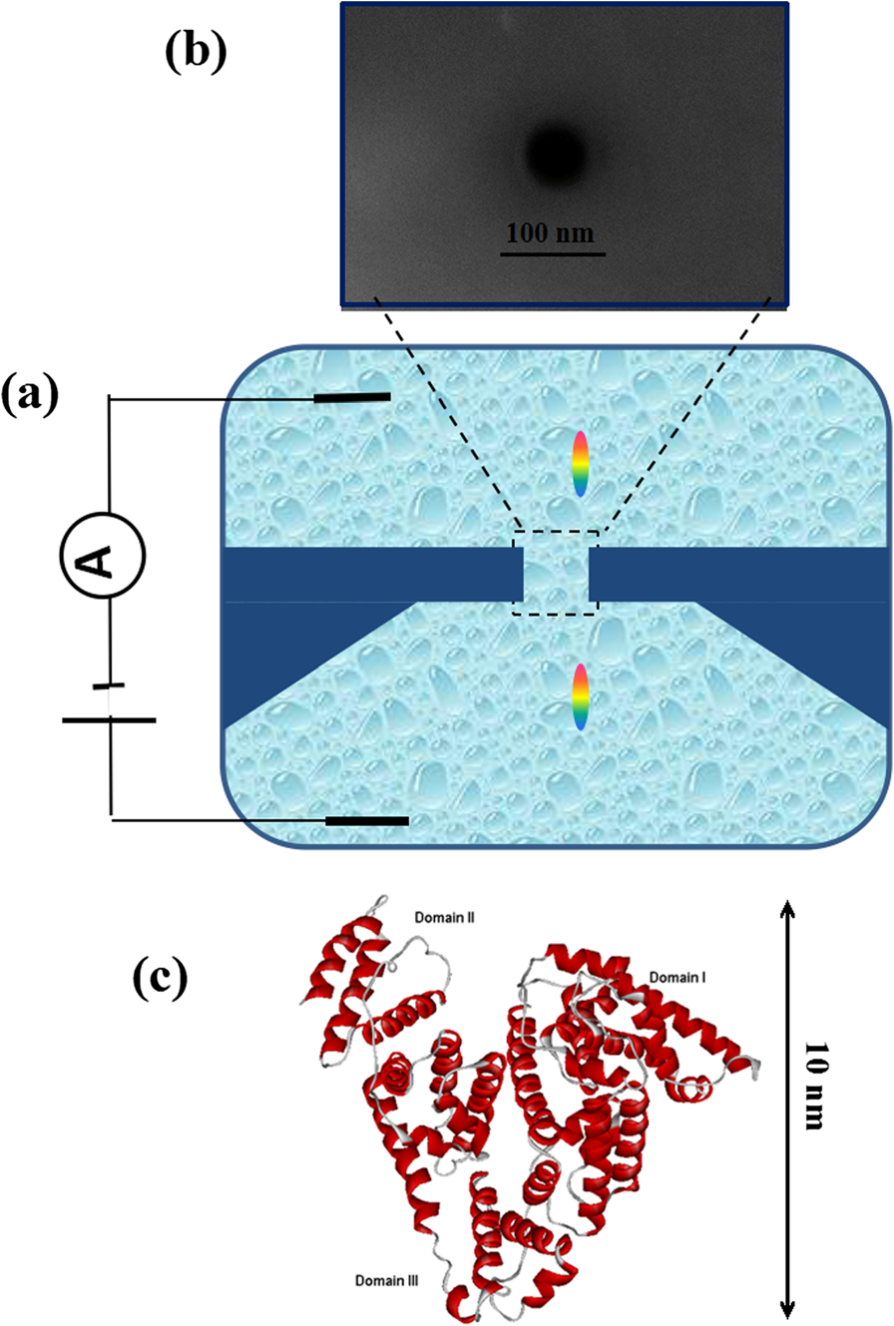
**Schematic illustrations of the microfluidic setup and nanopore detection. (a)** Schematic illustration of the microfluidic setup. A nanopore connects two compartments filled with an electrolyte solution (1 M/1 M KCl *cis*/*trans*), separated by a silicon nitride membrane. The application of an electric potential difference via two Ag/AgCl electrodes generates an ionic current through the pore. **(b)** A SEM image of approximately 60-nm nanopore fabricated by FIB, with a scale bar of 100 nm. **(c)** The schematic conformation of bovine serum albumin (BSA). Serum is a negatively charged globular protein with 583 residues and consists of three domains (I, II, III); the hydrodynamic diameter of the native state is about 10 nm measured with dynamic light scattering at neutral condition.

The nanopore used in our study was fabricated in freestanding 100-nm-thick silicon nitride membranes supported by a 300-μm-thick silicon wafer (Si 100) using focused ion beam (FIB) milling followed by feedback-controlled ion beam sculpting. The FEI Strata 201 (Hillsboro, OR, USA) was used with an acceleration voltage of 30 kV and ion current at 1 pA. A great variety of nanopore sizes were obtained in control of the ion dose and ion drilling time. The detailed process is referred to in previous studies [40]. The resulting pore was imaged by scanning electron microscopy (SEM). The pore diameter used in our experiment is about 60 nm, as shown in Figure 1b.

### Experimental setup

The schematic of the experimental setup is shown in Figure 1a. The nanopore-containing chip encapsulated with two PDMS films was immersed in ionic solutions, which was then divided into two isolated reservoirs; 1 M KCl salt solution was added into the two isolated reservoirs. Two Ag/AgCl electrodes were inserted into the reservoirs, respectively, and connected to a patch clamp amplifier (Axon Instruments, Axopatch 700B, Molecular Devices, Sunnyvale, CA, USA). The ionic current was filtered at 10 kHz and sampled using a 16-bit DAQ card (National Instruments, Austin, TX, USA) for a better signal-to-noise ratio, operated with homemade LabVIEW software. The whole fluidic device was put in a Faraday cage for shielding electromagnetic noise. In order to clean the chip and increase the hydrophilicity of nanopores, silicon nitride chips were soaked in piranha solution (30% H_2_O_2_/H_2_SO_4_ 1:3 (*v*/*v*)) for 30 min at 80°C and then rinsed with double-purified water. The dried chip is ready for nanopore experiments.

## Results and discussion

### Detection of protein translocations

**Figure 2:**
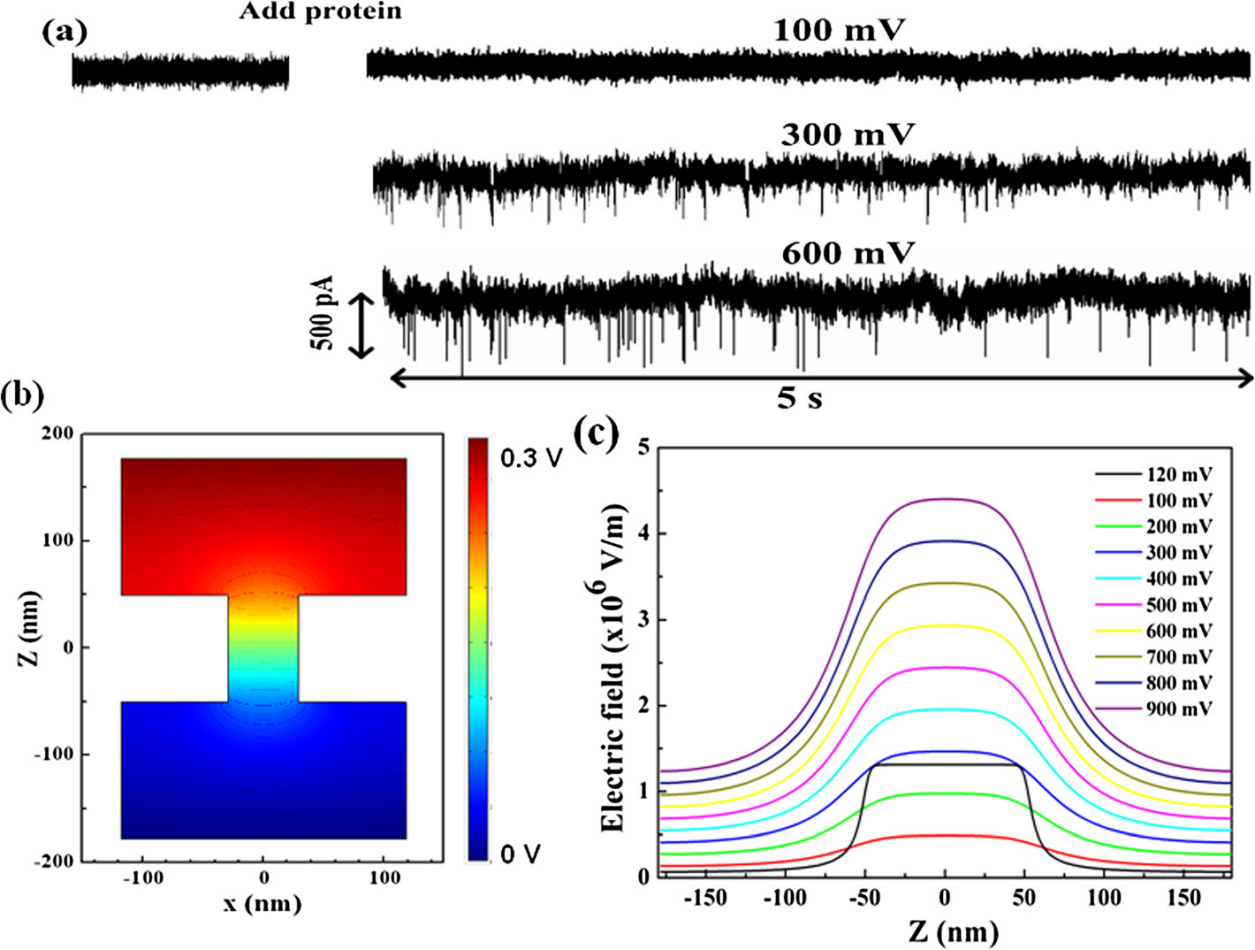
**Time recording of current traces, contour of electric field distribution, and electric field strength. (a)** Time recording of current traces recorded at 100, 300, and 600 mV of biased voltages. As a positive voltage was applied across the SiN membrane, a uniform, event-free open-pore current was recorded. The low noise in the baseline measurement allowed reliable identification of current blockages. After addition of protein in the *cis* reservoir, downward current pulses were observed at 300 and 600 mV. With the increase of voltages, the occurrence frequency of transition events was greatly improved. **(b)** Contour of electric field distribution of the cylindrical nanopore with a diameter of 60 nm as a function of biased voltages. **(c)** Electric field strength along the center axis of the pore.

When a positive voltage was applied across the silicon nitride membrane, a uniform, event-free open-pore current was recorded, as shown in Figure 2a. The low noise in the baseline measurement allowed reliable identification of current blockages. Subsequently, the protein was added to the negative reservoir and driven through the nanopore by a set of biased voltages. Unexpectedly, downward current pulses were not observed until a positive voltage of 300 mV was applied. With the increase of the voltage, the occurrence frequency of translocation events was greatly improved. However, the translocation events gradually disappeared when the voltage bias was below 300 mV.

It is well known that the electric field force is the main driving force for protein translocation through nanopores. Meanwhile, the hydrodynamic drag acting on proteins is opposite to the electrophoretic migration of proteins [8,10,15,41]. Thus, the negatively charged BSA (−18e at pH 7 in 1 M KCl) [29] experiences a competitive diffusion joined by electrophoresis and electroosmosis through the pore [35,41]. When the electric force is large enough to resist the drag forces acting on proteins, the protein is likely to enter the pore and pass through it. Thus, the driving force of the electric field is necessary for protein translocation through nanopores. However, compared with conventional small nanopores [15,29,42], the critical voltage (300 mV) for capturing proteins into the nanopore is higher in our studies. We expect that such a high threshold voltage is mainly associated with the larger dimension of nanopores. This scenario is confirmed by modeling the electric potential and field distribution of the nanopore using COMSOL Multiphysics [43], as shown in Figure 2b,c, where the nanopore is set with a diameter of 60 nm and a thickness of 100 nm. The distribution of the electric field is axially symmetric along the center of the nanopore, and most of the applied voltage drops across the pore (Figure 2b). For the same voltages, the electric field intensity in our pore is less than that of a small nanopore (10 nm). As the applied voltage increases to 300 mV, the electric field distribution is comparable to that of a smaller nanopore (10 nm) at the applied voltage of 120 mV. The electric field strength (*E*) along the center axis of the pore is also shown in Figure 2c. It is clear that the distribution of the electric field is approximately uniform in the pore while it is sharply decayed in the pore mouth. Thus, protein translocation through nanopores crosses over from almost purely diffusive to drift-dominated motion. There is a characteristic length scale that exists in the pore mouth for two forms of protein motion, which can be described by the Smoluchowski theory with a capture radius of r*[35,44]:

(1) $$r^{*}=\frac{d_{p}^{2}\mu \varphi }{8l_{p}D}.$$

Here *d*_p_ and *l*_p_ are the diameter and length of the pore, respectively, *μ* is the electrophoretic mobility, *D* is the protein diffusion coefficient, and *φ* is the biased voltage. This shows that the capture radius grows with the pore diameter and the biased voltages, and a bigger capture area can make more proteins trapped into the nanopore. Thus, a high throughput is expected in our nanopore device, which is also confirmed in our studies behind.

In addition, it is worth to mention the current noise in solid-state nanopores, which involves the 1/*f*-type excess noise and other contributions [45,46]. The 1/*f*-type noise is related to the fluctuation of charge carriers. As the voltage increases, the accelerated motion of charge carries will cause local ion aggregation in the nanopore, resulting in the increase of 1/*f*-type excess noise. It can be confirmed from the noise power spectra observed in our experiment (not shown here) and other experiments [45,46].

### Protein transport at the medium-voltage region

**Figure 3:**
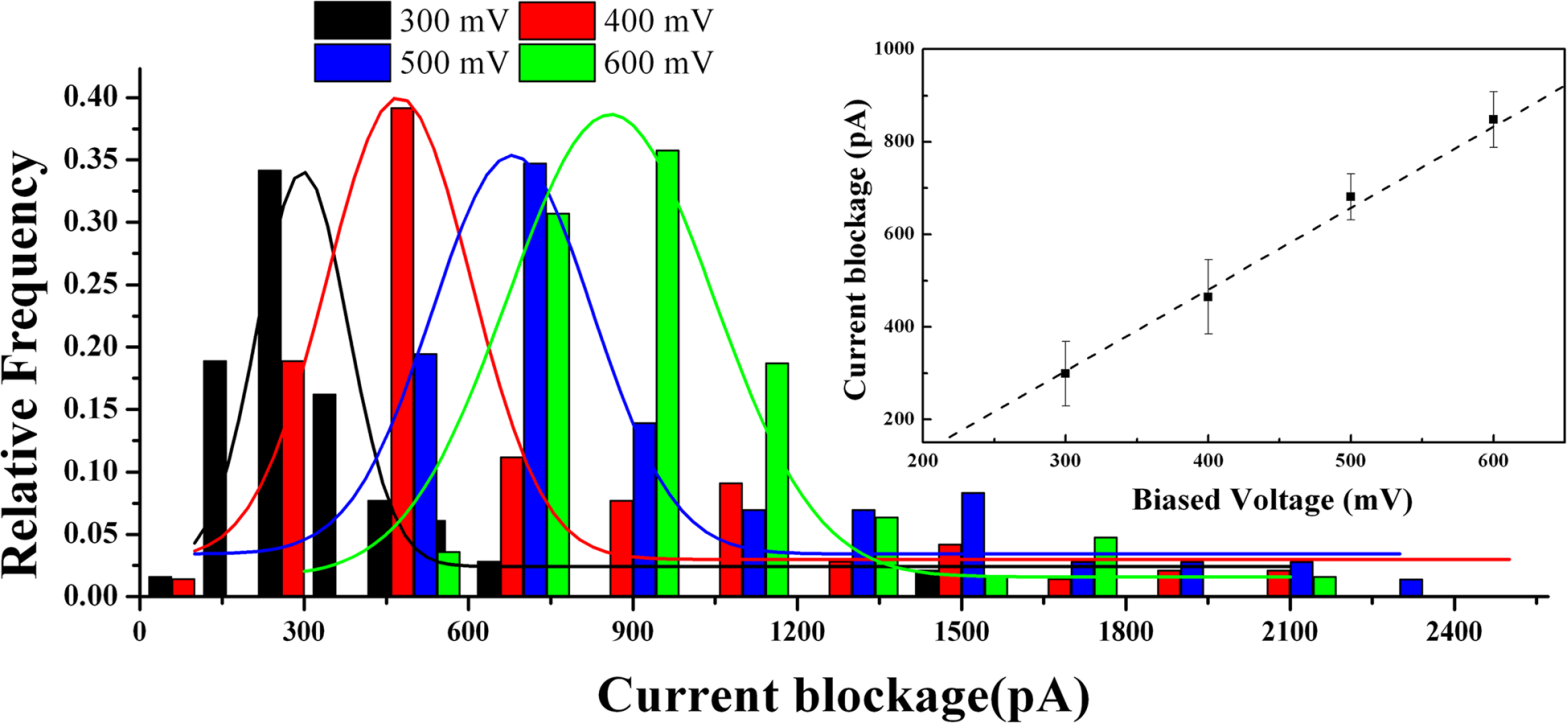
**Current blockage histograms as a function of applied voltage at medium voltages.** The histograms of current amplitude are normalized by fitting with Gaussian distribution; a linear increase of the means of current amplitude as a function of voltage can be clearly visualized in the inset. The numbers of translocation events at 300, 400, 500, and 600 mV are 102, 123, 156, and 160, respectively.

When the applied voltage is higher than 300 mV, a set of transient downward spikes appears, indicating the translocation of a single protein molecule across the pore. After confirming the ability of our large nanopore with a detectable signal-to-noise ratio, the voltage effects on the translocation signal have been studied in detail. Current blockage signals from individual molecular translocations can be characterized by the time duration (*t*_d_) and the magnitude of the blockage current (Δ*I*_b_). The histograms of the magnitude and dwell time of the transition events are characterized in our work. As shown in Figure 3, the amplitude distribution of blockage events at each voltage is fitted by a Gaussian mixture model. Based on the fitting curves, the peak values of the current blockages at 300, 400, 500, and 600 mV are 298, 481, 670, and 848 pA, respectively, which correspond to the most probable current drops induced by a single protein through the nanopore at varied voltages. The current amplitude linearly increases with the voltages, which yields a slope of 1.83 and an intercept of 265.51, as shown in the inset of Figure 3.

Based on the volume displacement of proteins in the electrolyte solution from the pore, the transient current blockage amplitude Δ*I*_b_ can be written as

(2) $$\Delta I_{b}\left(t\right)=-\frac{\sigma \varphi }{H_{\mathrm{eff}}^{2}}\Lambda \left(t\right)\left[1+f\left(\frac{d_{m}}{D_{p}},\frac{l_{m}}{H_{\mathrm{eff}}}\right)\right],$$

where *σ* is the solution conductivity, *φ* is the applied voltage between the electrodes, *Λ* is the excluded volume of a translocation molecule inside the pore, *H*_eff_ is the effective length of the nanopore, *d*_m_ is the diameter and *l*_m_ is the length of a particle molecule, *D*_p_ is the average diameter of a cylindrical nanopore, and $f\left(\frac{d_{m}}{D_{p}},\frac{l_{m}}{H_{\mathrm{eff}}}\right)$ is a correction factor that depends primarily on the relative geometry of the molecule and the pore [47,48]. Since the spherical-shaped protein is much smaller than the large nanopore, $f\left(\frac{d_{m}}{D_{p}},\frac{l_{m}}{H_{\mathrm{eff}}}\right)$ contributes little to the current drop. Thus, Δ*I*_b_ can be simplified as Δ*I*_b_(*t*) ~ *Λφ*, implying a linear dependence of the current blockade on the biased voltage. And the excluded volume of proteins in the pore can be calculated from the current drop. Based on the equation, the estimated volume of BSA in our experiments is about 260 nm^3^, which is very close to that of the native BSA structure (224 nm^3^) [29]. The volume change is less than 15%; thus, the unfolding of the protein destabilized by electric field forces can be ignored in the medium voltage from 300 to 600 mV, which appears in small nanopores due to the intensive electric field inside the pore [10,18].

**Figure 4:**
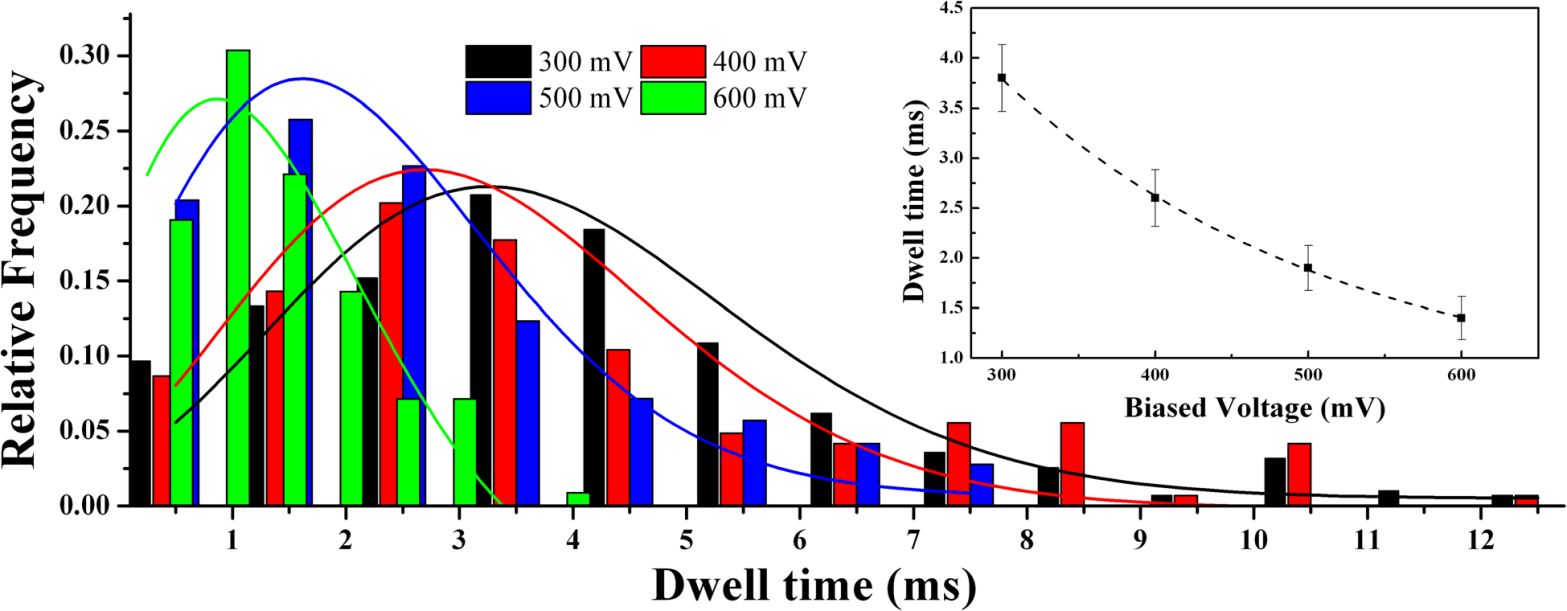
**Current blockage histograms as a function of applied voltage at medium voltages.** The histograms of time duration are fitted by exponential distribution. An exponential function of dwell time versus voltage is defined in the inset.

Meanwhile, the transition time of proteins also has been analyzed in our experiments. The current blockage duration *t*_d_ is regarded as the dwell time of a protein from the entrance to the exit of the nanopore. Majority of proteins quickly pass through the pore with less than 5 ms, typed as short-lived events. However, there is a small amount of blockage events with a prolonged transition time of tens of milliseconds, regarded as long-lived events, which are observed for protein translocations through small nanopores [31,32,47]. The distribution functions of transition times at each voltage have been analyzed in the present work. As shown in Figure 4, the histogram of dwell times shows an asymmetrical distribution, fitted by an exponential model. The mean transition times at 300, 400, 500, and 600 mV are 3.64, 2.45, 1.49, and 0.93 ms, respectively. An exponentially decaying function (*t*_d_ *~ e*^*−v*/*v*0^) is employed to fit the dwell time dependent on the voltage, as shown in the inset of Figure 4. Considering that the protein passes though the pore driven by electric forces (*F = Qφ*/*H*_eff_) with an average drift velocity *v = H*_eff_/*t* based on the Einstein relation, the effective diffusion constant can be estimated as *D*_eff_ *= K*_b_*Tφ*/*F ~* 10^−9^ cm^2^/s, which is far less than that in bulk (*D ~* 10^−7^ cm^2^/s) [29]. Hence, there are some interactions of protein-protein and protein-pore involved in the protein transition.

**Figure 5:**
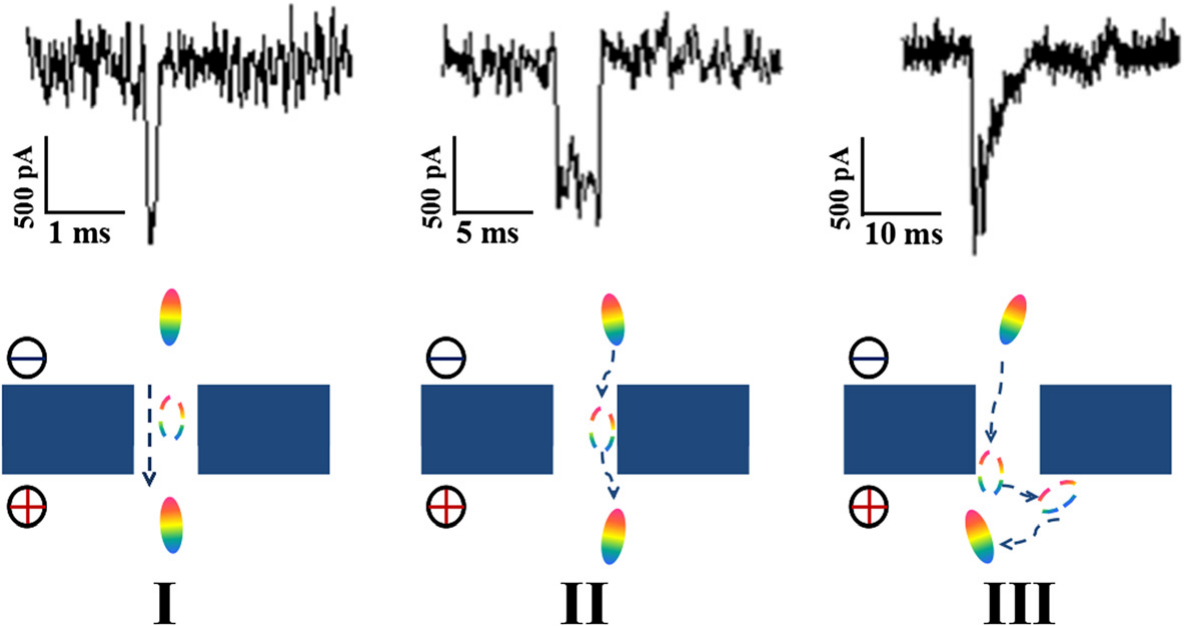
**Representative current blockades of translocation events at medium voltages.** In type I, the negatively charged protein will flash past the nanopore under strong electric forces within the nanopore. In types II and III, the protein is absorbed in the pore and around the pore mouth, respectively, for several milliseconds and then driven through the nanopore.

As mentioned above, the current blockage signals reveal the information of the size, conformation, and interactions of proteins passing through the nanopore. According to both *t*_d_ and *I*_b_, different types of discrete current blockades are characterized in Figure 5. For type I, the current signal has a typical spike shape with a deep intensity and a short dwell time. For type II, the current blockage turns to be rectangle with a similar amplitude but a long transition time. For type III, a distinct asymmetric and retarded current signal is observed with an even longer transition time. Usually, the negatively charged protein will flash past the nanopore driven by the strong electric force within the nanopore, giving the short-lived event as type I. However, given a protein with a high content of charged residues, a variety of electrostatic and hydrophobic interactions are involved in the liquid–solid interface between the protein and nanopore [31]. Once the protein is absorbed in the pore wall, the current signal will be blocked persistently, and it recovers till the protein is desorbed and impelled out the nanopore, showing as the long-lived events of types II and III. The type II event shows an abrupt restore, implying a very fast release of absorption. In contrast, the type III event shows a triangle-shaped signal and a longer restore period, implying a gradual release of absorption. Since the electric field (and thus the main driving force) within the nanopore is much stronger than that around the mouths of the nanopore (see Figure 2), it is reasonable to speculate that the absorption in the type II case is within the pore while that in type III is near the pore mouths. Owing to the decaying electric field in the pore mouth, there is a complicated equilibrium of adsorption and desorption involved between the protein and nanopore in type III. The absorption of protein to the nanopore wall also slows down the velocity of protein translocation, which accounts for the smaller diffusion constant *D* of proteins in the pore. In contrast with the prolonged dwell time from hundreds of milliseconds to several minutes obtained by small nanopores, the protein adsorption time is shortened and the frequency of the long-lived events is also decreased in larger nanopores. Especially, with the increase of the voltage, the adsorption phenomenon is gradually weakened by the enhanced driving force, and the velocity of protein transition is also speeded up. Thus, an exponential decrease of dwell time versus voltage is shown in Figure 3.

### Protein transport at the high-voltage region

**Figure 6:**
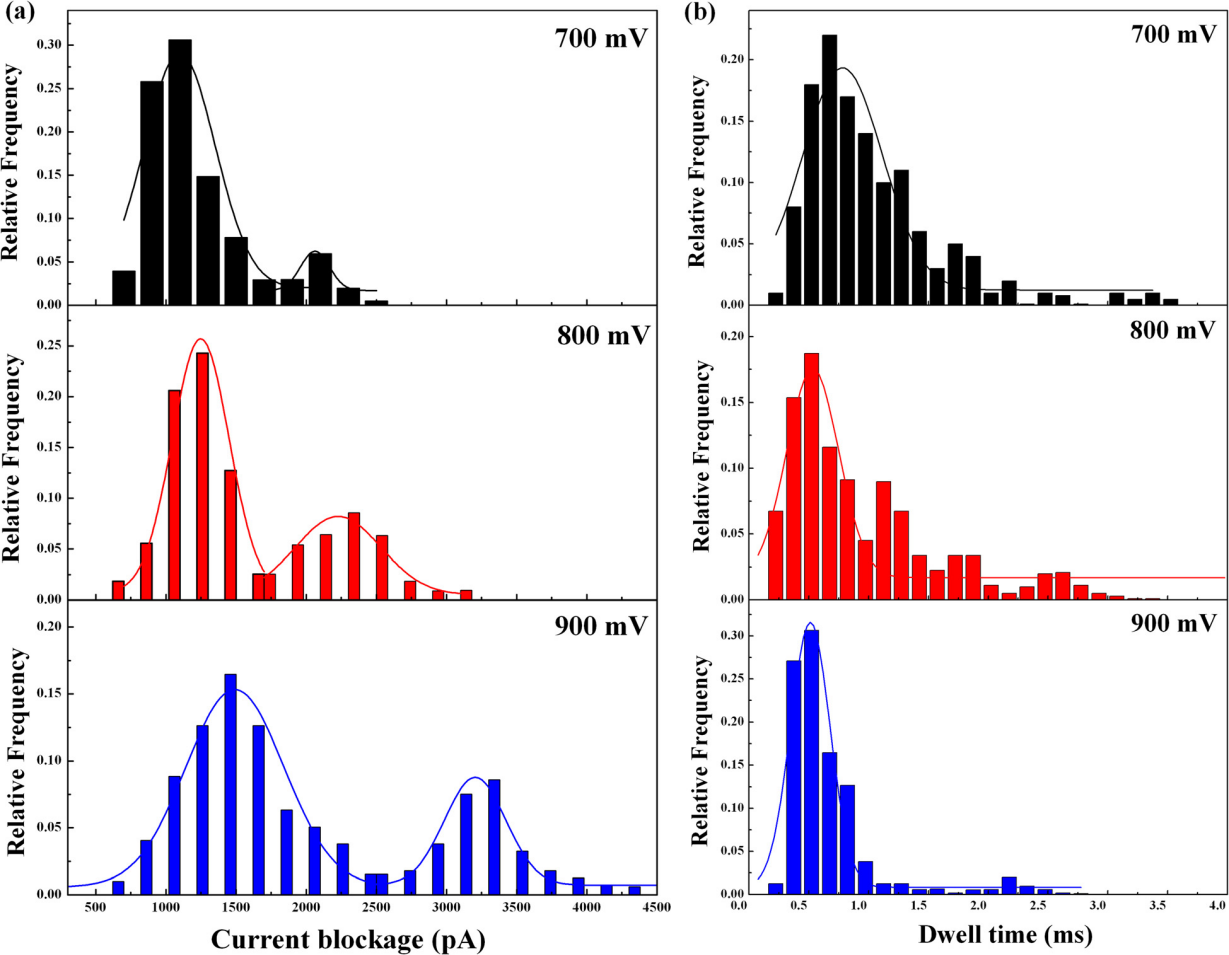
**Current blockage histograms as a function of applied voltage at high voltages. (a)** The histograms of current amplitude are normalized at voltages of 700, 800, and 900 mV. Multiple peaks with greater amplitude appear. **(b)** The histograms of time duration are fitted by Gaussian distribution at voltages of 700, 800, and 900 mV.

In the study of nanopore experiments, the applied voltage is one of the most critical elements for protein transports, which not only determines how fast protein translocations occur but also affects the interaction between proteins and nanopores [49]. In order to further investigate the voltage effect on protein translocations, the applied voltage was increased up to 900 mV. As expected, even a higher frequency of blockage events is detected at such high voltages. The histograms of the magnitude and dwell time of the translocation events at voltages of 700, 800, and 900 mV are shown in Figure 6. Different from the amplitude distribution with one main peak at the medium voltages, multiple peaks appear at high voltages in Figure 6a. Under these three voltages, the values of main peaks of the current blockages are 1,035, 1,229, and 1,500 pA, respectively, while the values of minor peaks are 2,058, 2,227, and 3,204 pA, respectively. Besides, the distribution of translocation times is also analyzed, as shown in Figure 6b. The most probable dwell times are significantly decreased to 0.75, 0.54, and 0.41 ms at the voltages of 700, 800, and 900 mV, respectively. The prolonged current events arising in medium voltages gradually decreased with increasing voltages. Therefore, besides the acceleration of protein translocations through the nanopore, the absorption interaction between the protein and nanopore is greatly suppressed at high voltages because the enhanced electric force can drag the protein away from the pore wall.

An intriguing question is the origin of the multiple peaks of current blockage that occurred at high voltages. First, a possible mechanism is related to the unfolding state of the protein disrupted by the enhanced electrical force, which is a common phenomenon observed in small nanopores [3,10]. Serum exhibits a heterogeneous charge distribution along its backbone, which allows for individual amino acids to be pulled in opposite directions. The electric force acting on each charged residue of the protein molecule is given by *F*_i_ = *Q*_i_ × *E*, where *Q*_i_ is the charge carried by each residue and *E* is the electric field strength [21], while the summation of *F*_i_ is the global driving force applied on the whole protein. The electric force acting on the protein is more than 10 piconewtons (pN) at high voltages above 700 mV. As proteins can be destabilized by elongation forces of several piconewtons based on the force spectroscopy measurements [50-52], the protein is potentially stretched into unfolding state with increasing voltages in the nanopore. Based on excluded volume values estimated from the main peaks at high voltages, the maximal volume change of protein is up to 50% in our high voltage experiments, which indicates that the protein has been stretched into an extended conformation by increased electric forces. Additionally, the excluded volume derived from the minor peak is about twofold of that from the main peak. The substantial growth of current amplitude is not merely the structural change of a single protein. Then we propose that the main peak with low magnitude is described by one protein (partial or full denatured state) entering the pore, and the minor peak with high magnitude is described by two molecules passing through the nanopore at the same time. The dimension of the nanopore is about five times as large as the protein, which allows multiple proteins to simultaneously pass through the nanopore. Especially, the stronger electric forces drive more molecules rapidly towards the nanopore. Thus, there is a higher probability of multiple molecules together entering into the pore at high voltages.

**Figure 7:**
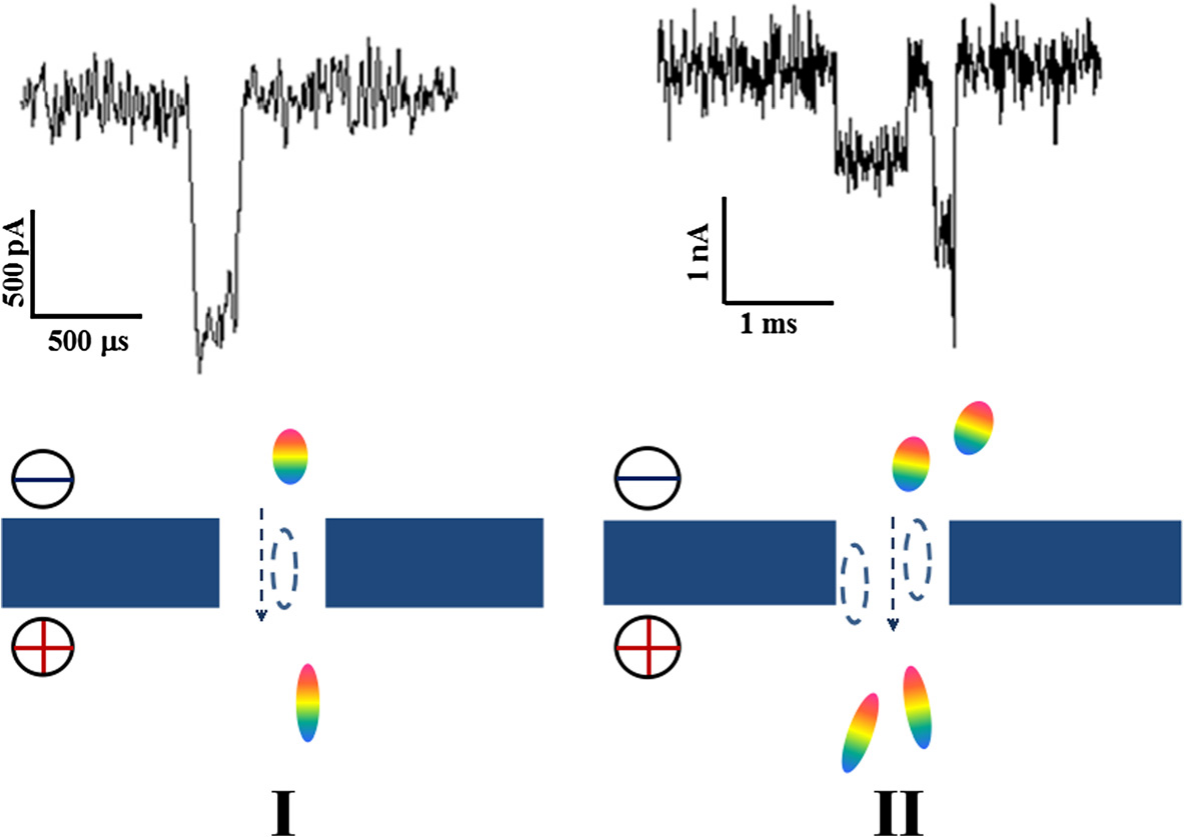
**Typical examples of translocation events at high voltages.** In type I, the negatively charged protein fast passes through the nanopore driven by the strong electric forces. In type II, a couple of molecules simultaneously pass through the nanopore.

Both types of protein transition events at high voltages have been defined, as shown in Figure 7. For type I, the event presents a short duration and greater amplitude, which suggest that the passing protein is stretched into a larger volume through the nanopore. For type II, the signal shows two blockage pulses. The current amplitude of the first current drop is half of that of the second while the duration of two events is similar with several milliseconds. In this case, a couple of proteins have been impelled into the nanopore simultaneously, which produces a double of current blockage. The current amplitudes of translocation events in the two types are quite different from each other. Nevertheless, the distribution of their transition times is overlapped in our work.

### Protein capture rates depending on voltages

**Figure 8:**
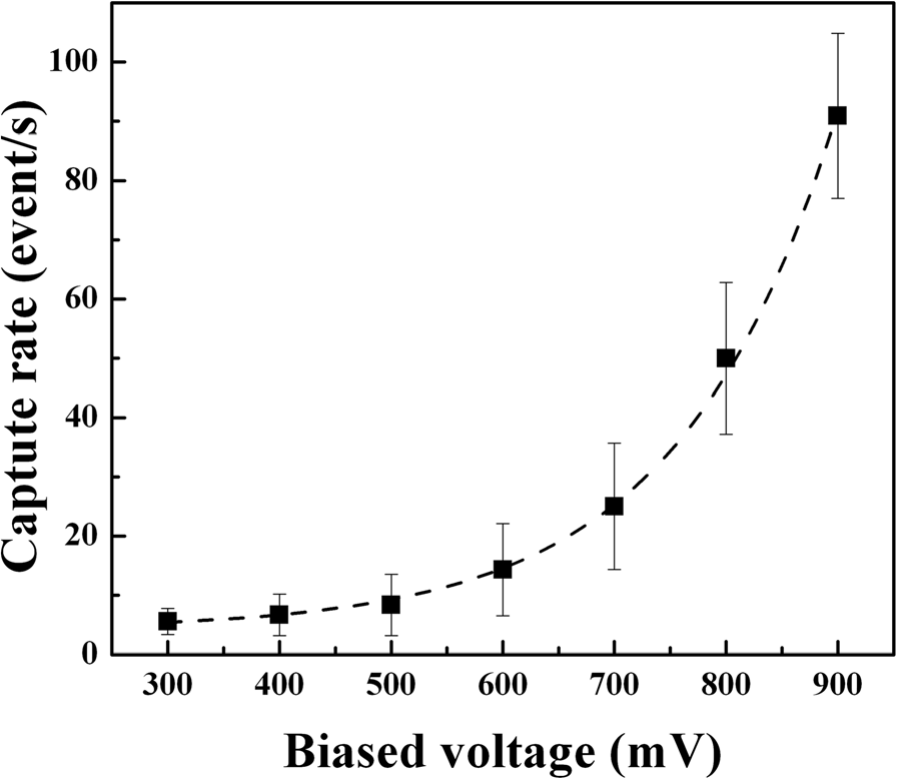
**The capture rate as a function of voltages.** The relationship of capture rate versus voltages is well fitted by an exponential function.

As described above, nanopore experiments on proteins are observed with long translocation time and low detected event rates at present. A barrier-limited transport is reported in small nanopores involving entropic fluctuation, protein absorption, and electroosmotic effects [3,16,48]. In our large nanopore, a large number of current blockage events are detected with varied voltages. The voltage dependence on the capture rate was analyzed in our work. As observed in Figure 8, the capture rate slowly increases at the medium voltages while it is sharply increased at high voltages. The whole trace of capture rate versus voltages is well fitted by an exponential function based on the Van’t Hoff Arrhenius law [3,16], which can be described as follows:

(3) $$R=R_{0}\exp\left(\left|V\right|/V_{0}\right)$$

Here *R*_0_ ∝ f^*^ exp(−U^*^/*k*_B_*T*) is the zero voltage capture rate controlled by an activation barrier U^*^ of entropic and electrostatic effect (f^*^ is a frequency factor). The ratio |*V*|/*V*_0_ is a barrier reduction factor due to the applied voltage. The potential *V*_0_ corresponds to the necessary applied potential to allow a charged protein to overcome the Brownian motion. From the fitted exponential function, we obtain *R*_0_ *=* 3.01 ± 1.1 Hz and *V*_0_ = 268 ± 8.9 mV*.* The voltage value is close to the threshold of 300 mV obtained in our measurement, which is necessary to drive the protein into the nanopore. It is known that the protein translocation through the nanopore is involved in the completion of the electroosmotic flow and electrophoretic mobility. The electroosmotic flow will suppress the penetration of the negatively charged proteins into silicon nitride pores, and its velocity increases with the electrical field. As the electroosmotic effect is dominant in small nanopores, the capture rate would decrease with the applied voltage increasing. However, an exponential increase of capture rate is observed as a function of voltages in our experiment. Thus, the electroosmotic effect is minor in our experiment with a large nanopore. With the increasing voltages, more protein is crowded at the pore entrance. Hence, the phenomenon of two molecules entering into the pore simultaneously occurs due to the high electric potential and large dimension of the nanopore.

## Conclusions

In summary, electrically facilitated protein translocation through a large nanopore has been investigated in our work. A large number of current blockage events are detected above the voltage of 300 mV. The distribution of the current magnitude and dwell time of the transition events are characterized as a function of applied voltages. Major proteins rapidly pass through the pore in a short-lived form, while minor long-lived events are observed with a prolonged time. With the increase of voltages, the current amplitude linearly increases while the dwell time is exponentially decreased. Meanwhile, the capture rate of proteins is greatly enhanced with an exponential growth. The protein absorption phenomenon and electroosmotic flow, which are dominant in small pores, are also compared in our work. These phenomena are weakened in large nanopores, especially at high voltages. Based on the excluded volume theory, the conformational change of proteins is estimated during the translocation process, and the unfolding of the protein stretched by intensive electric forces is confirmed at high voltages. The results show a new aspect of protein transport through a solid-state nanopore with a large size, which can provide more motivation for the development of nanopore devices as multifunctional sensors to analyze a wide range of biopolymers and nanomaterials.

## Abbreviations

BSA: bovine serum albumin; FIB: focused ion beam; pN: piconewton; SEM: scanning electron microscopy.

## Competing interests

The authors declare that they have no competing interests.

## Authors’ contributions

ZL, QL, and LW designed the protein translocation experiments through nanopores. LW carried out the protein translocation experiments and drafted the manuscript. LW, HL, and WZ participated in the statistical analysis. LW and CH participated in the nanopore fabrication. All authors read and approved the final manuscript.
